# Information and counselling on sexual health in cardiac and respiratory disease: description of community dwelling patients’ needs and reflections of health care professionals

**DOI:** 10.1186/s12939-025-02682-1

**Published:** 2025-11-17

**Authors:** Leonie Klompstra, Tommy Persson, Elin Klingvall, Anna Skoglund, Frida Särén, Tiny Jaarsma

**Affiliations:** 1 Department of Health, Medicine, and Caring Sciences, Linkoping University, Linkoping, 581 83 Sweden; 2 Knowledge Centre for Sexual Health, Västra Götalandsregionen, Kungsgatan 11, Göteborg, 411 18 Sweden; 3 Department of Cardiology, University Medical Center Utrecht, Utrecht, The Netherlands

**Keywords:** Sexual health, Well-being, Chronic condition, Respiratory disease, Cardiac disease, Healthcare professionals, Information need

## Abstract

**Background:**

Sexual health is an important aspect of overall well-being, encompassing physical, emotional, mental, and social dimensions. Despite its importance, many patients with chronic conditions, such as cardiac and respiratory diseases, experience challenges with sexual health and lack adequate information and support regarding sexual health from healthcare professionals (HCPs). Most studies on sexual health are conducted in clinical populations, but data on sexual health and information needs among community-dwelling cardiac and respiratory patients are scarce. Therefore, this study had two aims: (1) to describe and assess the experiences, needs and preferences among community dwelling patients with a cardiac and/or respiratory disease, regarding sexual health information and counselling; (2) to describe HCPs’ experiences and the conditions under which they provide information and counselling on sexual health.

**Methods and results:**

A cross-sectional design was used, including an online survey of patients with cardiac and respiratory diseases and focus groups with health care professionals involved in the care of patients with cardiac and respiratory disease. A total of 144 patients with cardiac and/or respiratory diseases participated in the survey, with 85% reporting that their sexual health affected their well-being. Only 7% said they received information on sexual health from healthcare providers, while 84% expressed a desire for information or counselling regarding sexual health. Ten HCPs participated in the focus group, revealing five themes related to providing sexual health information and counselling: personal biases, organizational factors, societal norms, knowledge and experience, and the need to break mutual silence. They highlighted the importance of professionalism, respect, and clear communication in addressing sexual health among patients.

**Conclusion:**

This study highlights a significant gap between the sexual health needs of patients with cardiac and/or respiratory diseases and the information provided by HCPs, with most patients desiring more support. HCPs should proactively address sexual health in patients with cardiac and respiratory diseases. Overcoming barriers such as personal biases, organizational constraints, and societal norms requires respectful communication, professional training, and supportive healthcare structures.

**Supplementary Information:**

The online version contains supplementary material available at 10.1186/s12939-025-02682-1.

## Introduction

 Sexual health is fundamental to the overall health and well-being of individuals, couples and families according to the definition of the World Health Organization (WHO) [1]. Sexual and reproductive health is a state of physical, emotional, mental, and social well-being in relation to all aspects of sexuality and reproduction, not merely the absence of disease, dysfunction, or infirmity [2]. The ability to achieve sexual health and well-being depends on several factors, one of which being the ability to access comprehensive, good-quality information about sex and sexuality [1]. The sexual health of people with a chronic condition can be affected by the symptoms and treatments of their condition [3, 4]. Chronically ill individuals are 2–6 times more likely to be affected by sexual dysfunction than healthy people of the same age and gender [4]. For example, among people with cardiac disease, the proportion experiencing a sexual dysfunction, such as erectile dysfunction (ED), is twice as high as the general population [5–7]. In patients with respiratory diseases (e.g. Chronic Obstructive Pulmonary Disease (COPD) and asthma) sexual dysfunction is also frequently reported and associated with reduced well-being [8, 9].

The reasons for sexual problems among people with a chronic illness are sometimes attributed to side-effects of medication that can cause ED, vaginal dryness or decreased libido [10]. However, there is still no clarity on the relationships between many cardiovascular drugs and sexual problems [11]. Other reasons for sexual dysfunction might be fear and worry about a new myocardial infarction or exacerbation of physical or psychological symptoms related to the disease or its treatment (e.g. surgery), or an impact on sexual desire or relationships [12].

A scoping review [4] concluded that people with chronic diseases want their healthcare providers (HCPs) to initiate discussions about sexual concerns and treat them respectfully. Patients with cardiovascular diseases described the need for information about the risks that may exist during sexual activity and advice on whether it is possible to have sex after a cardiac event or treatment. Providing patients with cardiac and respiratory diseases with information, advice and support regarding sexuality can be important for their quality of life [13].

At the same time a plethora of studies shows that although health professionals are aware of the importance of discussing sexual function and sexual activity, they refrain from doing so [14–16]. Concerns include the fear of “opening up a can of worms,” limited time, resources, and training, doubts about one’s knowledge and abilities, apprehension about causing offense, personal discomfort, and a lack of awareness of sexual issues [15–17].

Most studies on sexual health and the need for information and counselling are conducted in clinical populations within specialized care settings, but data on sexual health and information needs among community-dwelling cardiac and respiratory patients are scarce.

Therefore, this study had two aims: (1) to describe and assess the experiences, needs and preferences among community dwelling patients with a cardiac and/or respiratory disease, regarding sexual health information and counselling; (2) to describe HCPs’ experiences and the conditions under which they provide information and counselling on sexual health.

## Method

A cross-sectional study design was used, including an online survey of patients with cardiac and respiratory diseases and a focus group with HCPs involved in the care of patients with cardiac and respiratory disease.

### Procedure

Participants for the survey were recruited via patient organizations, clinical outpatient departments, hospitals, primary healthcare clinics, and Swedish national heart and lung patient associations. Flyers with QR codes were distributed to members of Swedish national heart and lung patient associations and posted in outpatient clinics. Additionally, electronic flyers, with a clickable link to the survey, were posted on social media in Sweden and participants could choose to complete the questionnaire in Swedish or English.

All survey data were collected via patient self-report and data were collected anonymously. IP addresses were not recorded. Ethical approval was obtained (Dnr: 2023-06695-01).

The HCPs were recruited via a broad invitation sent out by e-mail to all healthcare providers in primary and specialised care, working with cardiac and/or respiratory patients, in one region in Sweden (Region Västra Götaland). The invitation included details about the study and a link to a registration site. All HCPs provided written informed consent before participating. The session lasted 180 min and was conducted in a conference room outside their workplace to ensure privacy.

The Consensus-Based Checklist for Reporting of Survey Studies (CROSS) was used for the data from the patient survey, the consolidated criteria for reporting qualitative research (COREQ) guidelines were used for the focus groups with the HCPs.

### Measurements

The 25-question patient survey was developed based on literature and previous international surveys on sexual health in cardiac patients [18, 19]. The survey included questions on sexual health, as well as information and counselling participants had received regarding sexual health (e.g. when, what, where, from whom), and their need for information and counselling. To capture the patients’ perspectives on what is important for HCPs to consider when providing information and counselling about sexual health, an open-ended question was added (Supplementary material 1). We also collected demographics and information on participants’ health condition through the survey.

The focus group was moderated by two of the authors (TP and AS) and co-moderated by two other authors (EK and FS). All had extensive experience with focus group interviews, and extensive experience working with sexual health. The co-moderators made observations, took notes, and helped to identify gaps, contradictions, and explore reasoning paths during the focus group session that the moderators might overlook.

The focus group was conducted using an interview guide. To extract more details, further explanations, and examples, the moderator used probes and asked follow-up questions (e.g., Do you all agree with this? Can you give examples? What do the rest of you think about this). The focus group was audio-recorded and transcribed verbatim by a professional transcribing firm. The transcript was scrutinized and corrected for any errors by the authors to ensure high quality and accuracy.

### Data analysis

Means and standard deviations were calculated for continuous data, and absolute numbers and percentages were computed for nominal variables.

Differences between genders and age groups were assessed. As only one patient identified as transgender, we assessed the differences between patients who identified as male versus female by Chi-square test. Patients younger than 65 years were compared with patients 65 years or older with Chi-square tests, these are reported only when differences were found.

Qualitative data from the open-ended question in the questionnaire were analysed separately with inductive content analysis, whereby meaningful units were coded, and subcategories were developed based on these codes. As the data of the open-ended question in the questionnaire was not rich, in this analysis these subcategories became categories.

The focus group data were analysed using qualitative content analysis, following the methodology outlined by Graneheim and Lundman (2004) [20] and further elaborated by Graneheim, Lindgren, and Lundman (2017) [21].The transcripts were initially read multiple times by all authors to gain a comprehensive understanding of the content and to assess its relevance to the study’s aim and research questions. Subsequently, relevant segments of the text were identified as meaning units, which were then condensed and assigned codes.

To enhance the trustworthiness of the analysis, this coding was conducted independently by each author. Special attention was given to the coding performed by LK, who had not been involved in conducting the focus group interviews, to provide an external perspective. The codes were then grouped into sub-themes, which were further abstracted into overarching themes that captured the latent and manifest content of the data.

The analytical process was iterative and reflexive, involving constant comparison and revisiting of earlier stages to ensure coherence and depth in theme development. Discussions among the authors were integral at each stage, with consensus being reached through collaborative interpretation. In the final phase, the raw data were revisited to validate and refine the thematic structure. Field notes from the focus group interview were also consulted throughout the analysis to support the contextual understanding of the data. Preliminary findings were regularly reviewed and revised in collaborative discussions among the authors.

## Results

In total, 144 patients with a cardiac and/or respiratory disease completed the online survey. The mean age was 65 (± 13), 59% identified as male and one person identified as transgender. Almost half of the patients reported having hypertension (45%, *n* = 65). The next most commonly reported diseases were myocardial infarction (35%, *n* = 49), atrial fibrillation (28%, *n* = 40) and heart failure (23%, *n* = 33) (Table 1a).

**Table 1:** Demographics and clinical variables of 144 patients with cardiac and/or respiratory disease(s)

	Total	Male 59% (*n* = 84)	Female 41% (*n* = 59)	Age < 65 years 45% (*n* = 64)	Age ≥ 65 years 55% (*n* = 77)
**Age**	65±13	66±12	63±15	52±9	75±6
Conditions*					
Hypertension	45% (*n* = 65)	43% (*n* = 36)	49% (*n* = 29)	42% (*n* = 27)	48% (*n* = 37)
Myocardial Infarction	34% (*n* = 49)	45% (*n* = 38)	17% (*n* = 10)	31% 8n = 20)	35% (*n* = 27)
Atrial fibrillation	28% (*n* = 40)	25% (*n* = 21)	32% (*n* = 19)	20% (*n* = 13)	35% (*n* = 27)
Heart Failure	23% (*n* = 33)	17% (*n* = 14)	32% (*n* = 19)	20% (*n* = 13)	23% (*n* = 18)
Angina Pectoris	17% (*n* = 24)	23% (*n* = 19)	9% (*n* = 5)	19% (*n* = 12)	14% (*n* = 11)
Lung disease	15% (*n* = 21)	11% (*n* = 9)	20% (*n* = 12)	14% (*n* = 9)	16% (*n* = 12)
Arrythmia	14% (*n* = 20)	12% (*n* = 10)	15% (*n* = 9)	13% (*n* = 8)	13% (*n* = 10)
Cardiac Arrest	6% (*n* = 9)	10% (*n* = 8)	2% (*n* = 1)	11% (*n* = 7)	3% (*n* = 2)
Valvular Disease	6% (*n* = 9)	6% (*n* = 5)	7% (*n* = 4)	6% (*n* = 4)	6% (*n* = 5)
Congenital Heart Disease	6% (*n* = 8)	5% (*n* = 4)	7% (*n* = 4)	11% (*n* = 7)	1% (*n* = 1)
Thrombosis	5% (*n* = 7)	4% (*n* = 3)	7% (*n* = 4)	2% (*n* = 1)	8% (*n* = 6)
Stroke or Transient Ischaemic Attack (TIA)	6% (*n* = 8)	2% (*n* = 2)	10% (*n* = 6)	6% (*n* = 4)	5% (*n* = 4)
Aneurysm	4% (*n* = 5)	5% (*n* = 4)	2% (*n* = 1)	5% (*n* = 3)	3% (*n* = 2)
Sleep Apnoea	2% (*n* = 3)	1% (*n* = 1)	3% (*n* = 2)	0%	4% (*n* = 3)
**Cardiac implanted devices or treatments**					
Pacemaker	11% (*n* = 16)	13% (*n* = 11)	9% (*n* = 5)	9% (*n* = 6)	13% (*n* = 10)
Defibrillator	8% (*n* = 12)	6% (*n* = 5)	10% (*n* = 6)	13% (*n* = 8)	4% (*n* = 3)
Cardiac Surgery	11% (*n* = 16)	15% (*n* = 13)	5% (*n* = 3)	9% (*n* = 6)	12% (*n* = 9)

*More than one answer possible

In total, 85% of the patients (*n* = 123) reported that their sexual health affected their well-being with 48% (*n* = 70) reporting they were affected to a large extent. More men than women reported that their sexual health affected their well-being (25% vs. 6%, p-value = 0.02). Furthermore, 73% of the patients reported that their disease affected their sexual health negatively (*n* = 105), while women reported more often that their disease did not affect their sexual health (36% vs. 18%, p-value < 0.01).

### Received information and counselling

In total, 7% of the patients (*n* = 10) reported having received information on sexual health from HCPs, primarily regarding medication side effects (*n* = 6), and most received this information from their doctor (*n* = 7). Patients who received information or advice on sexual health outside of healthcare (13%, *n* = 19), most found this information on the Internet (59%, *n* = 10). In total, 19% of the participants (*n* = 27) reported that they were able to ask their HCPs questions about sexual health.

### Wanted to receive information and counselling

Of the total number of patients, the majority (84%, *n* = 112) reported wanting or needing information or counselling on sexual health. More men reported to need information or counselling compared to women (87% vs. 62%, p-value < 0.01).

**Fig. 1:**
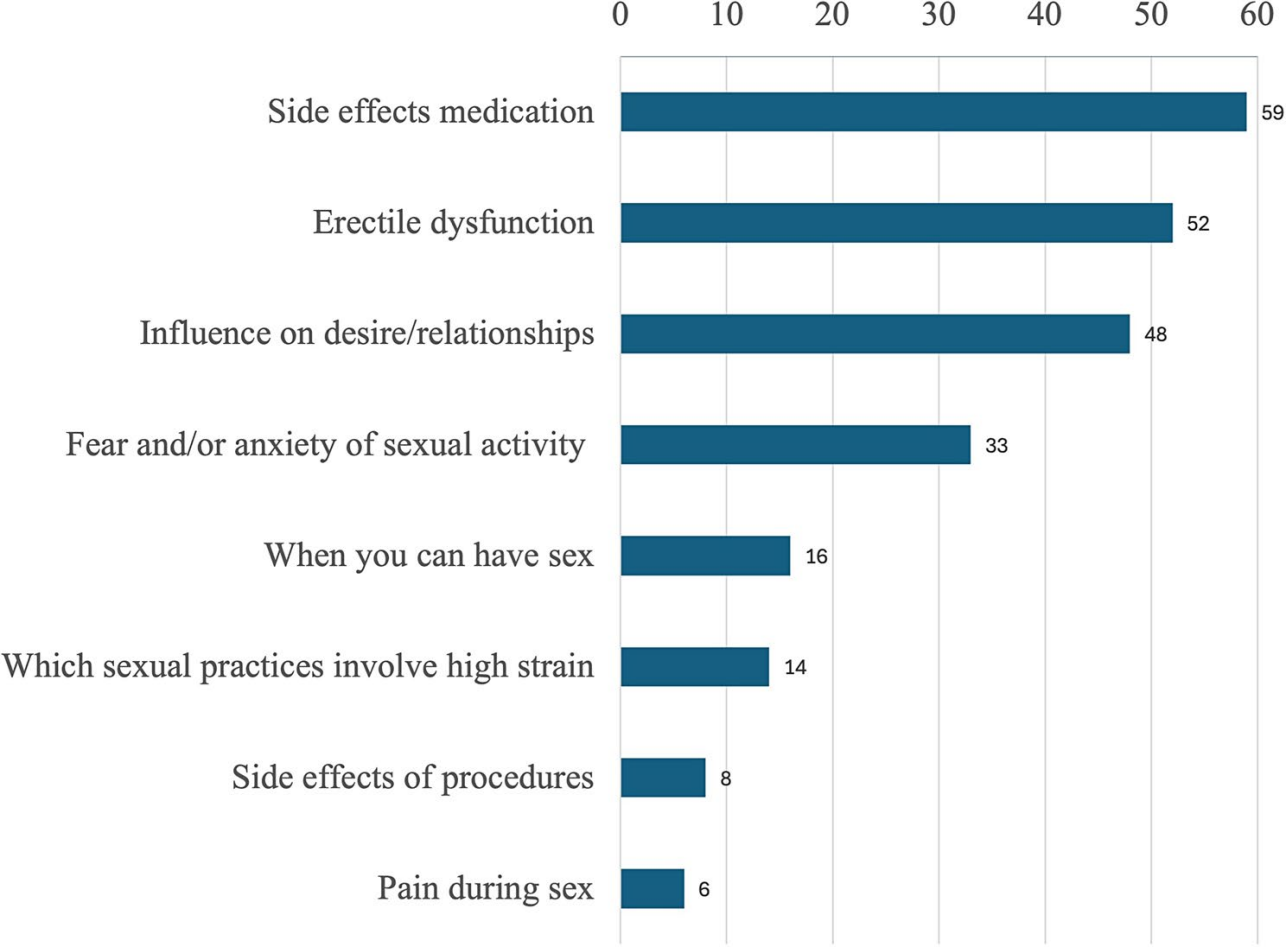
Percentage of patients interested in receiving information and counselling on various sexual health topics

Approximately half of the patients wanted information regarding side effects of medication (59%, *n* = 85), erectile dysfunction (52%, *n* = 69) and the influence of their disease on desire and their relationships (48%, *n* = 69). One-third of the patients would like to receive information on fear and/or anxiety of sexual activity (33%, *n* = 48) (Fig. 1). Most men wanted information on erectile dysfunction (77%) and woman on pain during sex (14%).

More than half of the patients wanted information on sexual health in connection with diagnosis (51%, *n* = 74), at annual check-up (51%, *n* = 74) or during medication prescription (58%, *n* = 83). Patients who were younger than 65 years more often wanted information on sexual health during follow-up compared to participants 65 years or older (39% vs. 27%, p-value < 0.01) (Table 2).

**Table 2:** When, from whom, where patients would like to receive information on sexual health and the preferred way of delivery on this information

When do patients want to receive information on sexual health*	
In connection with medication prescription	58% (*n* = 83)
During annual check-up	57% (*n* = 82)
In connection with diagnosis	51% (*n* = 74)
During follow-up visit for medication	39% (*n* = 56)
During follow-up visit after surgery	27% (*n* = 39)
Before surgery	6% (*n* = 9)
Patients would like to receive information from*:	
Doctor	79% (*n* = 113)
Nurse	60% (*n* = 87)
Social worker or psychologist	38% (*n* = 54)
Physiotherapist	15% (*n* = 21)
National health service line (1177) chat or phone	8% (*n* = 12)
I don’t know	8% (*n* = 12)
Occupational therapist	8% (*n* = 11)
Dietician	5% (*n* = 7)
Where patients would like to receive information*	
Hospital	77% (*n* = 107)
Primary care	71% (*n* = 99)
Rehabilitation center	32% (*n* = 44)
Preferred delivery mode*	
Conversation with HCPs	79% (*n* = 111)
Possibility to receive information and advice at different times from different sources	43% (*n* = 60)
Brochure	38% (*n* = 54)
Patient organisation	35% (*n* = 49)
National health service line (1177)	27% (*n* = 38)
Peer groups	12% (*n* = 17)
Information film	10% (*n* = 14)
App	10% (*n* = 14)

* More than one answer possible

HCP: Health care professional

Most patients would wanted information from either a doctor (79%, n 0 113) or a nurse (60%, *n* = 87) and in the hospital (77%, *n* = 107) or their primary care team (71%, *n* = 99) (Table 2).

Most patients wanted information on sexual health through conversations with their health care professionals (79%, *n* = 111). Other reported delivery means included a brochure (38%, *n* = 54), through patient organisations (35%, *n* = 49) or through an online health portal called 1177 (27%, *n* = 38) (Table 2). More women than men would like to receive information on sexual health through a brochure (48% vs. 31%, p-value = 0.04). Younger patients were more likely to want information and counselling on sexual health from HCP’s (91% vs. 71%, p-value < 0.01) or through a Mobile Application (81% vs. 4%, p-value < 0.01) compared to patients 65 years or older.

### What is important to consider as a HCPs when providing information or counselling on sexual health?

We are ordinary people with perhaps un-ordinary diseases. Sexual health is just as important to us as it is to healthy people.

### Patients’ perspectives on what is important for HCPs to consider

The results from the free-text answers in the patient survey revealed four categories: (1) professionalism and knowledge; (2) respect and sensitivity; (3) person-centered approach; (4) clear communication.

1. **Professionalism and knowledge**: Patients reported that it is important for HCPs to share experiences from others and provide detailed information. HCPs should be well-informed, knowledgeable, and able to discuss sexual health without embarrassment. They should also be aware of the impact that medications can have on sexual health and communicate this clearly.
2. **Respect and sensitivity**: Patients expressed that HCPs should have respectful and professional conversations with patients and be sensitive to the individual’s conditions and treatment. HCPs should be empathetic, listen actively and not avoid discussing sexual health.
3. **Person-Centered Approach**: Addressing sexual health should be done at the right time, allowing for individual processing. Patients expressed that HCPs should be aware of the diverse experiences and needs of patients and should include partners in education and support.
4. **Clear Communication**: HCPs should be straightforward and honest. They should be prepared to discuss potential problems and solutions openly and clearly and invite patients to ask questions about sexual health. Encouraging open dialogue and ensuring patients feel safe to discuss their sexual health concerns without judgment is important.

### Health care professionals’ experiences of and conditions for providing information and counselling on sexual health

In total 10 HCPs participated in the focus group; the mean age was 44 (± 8) and 8 were female. Two HCPs received training in sexual health, and they had a median time experience treating patients with cardiac and respiratory disease of 15 years (IQR 1–25) (Table 3).

**Table 3:** Demographics of healthcare professional participating in the focus group (*n* = 10)

	Total *n* = 10
**Age (Mean±SD)**	44±8
**Female sex**	80%
**Degree**	
Specialised nurse in cardiology	40%
Registered nurse	30%
Physiotherapist	20%
District nurse	10%
**Workplace**	
Cardiac clinic	60%
Primary healthcare center	20%
Physiotherapist clinic	20%
**Experience with cardiac and/or respiratory patients** (Years) (Median, IQR)	15 (9–20)
**Received training in sexual health**	20%

Our analysis identified five themes regarding HCPs experiences of and conditions for providing information and counselling on sexual health related to their reflections on the results of the survey: (I) It is person-dependent, (II) Organizational prerequisites, (III) Sexual norms and views, (IV) Knowledge and experience, and (V) Breaking the mutual silence (Table 4).

**Table 4:** Themes and subthemes on experiences of and conditions for providing information and counselling on sexual health

Themes	Subthemes
I. It is person-dependent	Age
	Notions about culture and ethnicities
	Specific group of patients and diagnose
	Patients’ needs
	Person-centred care
	Presence of relatives and/or partners
II. Organizational prerequisites	Availability and accessibility
	The importance of the situation and arena
	Time
	Information material
	Routines and guidelines
	Coworkers, colleagues, and the team
	Who should address sexual health
	Organization and workplace
III. Sexual norms and views	How HCP perceive the subject (sexual health)
	How patients perceive the subject (sexual health)
	Sexuality in society
IV. Knowledge and experience	Education and knowledge
	Knowledge of the connection (between CVD and sexual health)
	What HCP need and do not need
	Security, trust, and experience
V. Breaking the mutual silence	The relationship between patient and HCP
	Perceived role of the HCP
	Resistance, obstacles, and barriers
	Making the subject talkable/breaking the silence
	Asking questions several times
	Signalling
	Professionalism
	How and when does the conversation happen

#### Theme I: it is person-dependent

Providing information and counselling on sexual health was described as conditional, based on who the individual patient was assumed to be, i.e., based on notions about patients’ identities and social positions such as gender, sex, age, and cultural background. HCPs had different perceptions of which patients wanted or needed to be asked about their sexual health, and there were differences in which patients participants preferred to discuss sexual health with.

HCP 1: “So what did you conclude, did some patients receive more questions than others?”

HCP 2: “A lot depended on the person.”

HCP 3: “Yes, exactly.”

Patients age and sex were factors that could be especially relevant, one HCP remarked that they had never talked to a woman about sexual health despite being a woman herself. A shared experience of the participants in the focus group was that sexual health was often reduced to questions about the impact of medication or treatment on erection, which meant that mostly men were asked and informed. Being the same age as patients was described as difficult, but opinions differed on whether it was easier to talk with younger or older patients than oneself.

… It is different depending on the age of the patient and we had very different preferences [on who we prefer to talk to]….

Patients cultural background was said to relate to their attitudes to sexuality, which could affect HCPs likelihood of asking patients, with a perceived ethnic background different from their own, questions about sexual health. There was no consensus on how relatives and partners should receive information and counselling. Experiences in the focus group differed regarding whether relatives and partners usually accompany patients or not. Some HCPs argued that relatives and partners could harbour similar fears and questions as patients and should therefore be offered information and counselling independently. Others stated that healthcare is not responsible for relatives and partners. Opinions differed on whether partners or relatives could influence how easy or difficult it was for HCPs and for patients to talk about sexual health.

Another factor that was said to influence if and how HCPs address sexual health was the imagined impact of the diagnosis or treatment on the patient’s sexuality.

Then we [as HCPs] can imagine that there is a worry about whether I [as a patient] would die the next time I have sex.

Some conditions, such as a heart attack or chest pain, were assumed to have a major impact on sexuality and cause an associated fear of death with sexual activity in patients. This meant that it was perceived to be both more important and more difficult to talk about sexual health.

#### Theme II: organizational prerequisites

Addressing sexual health and meeting patients’ needs and wants regarding information and counselling were described by HCPs as dependent on several organizational factors. Factors such as time-constraints, support or lack of support from management, the physical and social environment of healthcare, a general lack of routines and guidelines, staff competence and collaboration, as well as the availability and accessibility of individual clinics, were all considered influential.

At work, organizationally, we are not used to addressing that [sexual health].

Another request from HCPs concerned tools and material developed for patients that would assist and, in some ways, compensate for lack of organizational prerequisites. This included information material, handouts and posters.

HCP 1: “A poster or something can be enough, where it says, “we talk about sexual health as part of the general health”.”

HCP 2: “Dare to ask.”

HCP 3: “Yes, exactly,”

HCP 4: “That would be great.”

Such material, according to HCPs, had to appear professional to signal the importance of the subject to patients and the organization.

#### Theme III: sexual norms and views

The HCPs argued that their own and patients attitudes toward addressing sexual health in healthcare interactions were in part influenced by societal and personal views on sex and sexuality and by sexual norms. Attitudes of both patients and HCPs were described as complex and partly contradictory. Sexual health was described as engaging, an important subject, taboo and differently charged in theory and practice. Patients’ attitudes to sexuality were considered something that HCPs needed to account for in healthcare meetings dealing with sexual health.

Our effort has to be tremendously larger than for the patient [since] they enter the room bringing with them so many societal taboos, so we have to level up […].

HCPs views differed on whether the subject was more, equally, or less emotionally charged than other potentially private or personal topics that might be addressed in interactions with patients.

But, that is also something that was imposed on you when you were little, that this is a very private subject. That is not something that you can just erase.

#### Theme IV: knowledge and experience

Differences in knowledge of and experiences with sexual health were said to be important for HCPs willingness and ability to meet patients’ needs and wants for sexual healthcare. In the focus group, questions were raised among the HCPs about whether they possessed enough knowledge to provide adequate care and whether their professional education had offered sufficient prior knowledge on the topic.

I am just trying to figure out what questions [about sexual health] I would have been able to answer, […] if someone asks then you actually have to answer and if you don’t know then I think the best thing is to say that you don’t know.

HCPs discussed if it is enough to create permission and opportunity for patients to address potential sexual health issues or if HCP must possess expert knowledge to be able to answer all conceivable questions patients have.

There are quite a few of us who don’t have a grasp of the situation, any situation, so absolutely, just daring to talk about it and sort of raise it on the agenda [is important], yes, but somewhere [we need to] raise the bar to actually have, they do want knowledge and what can I say then? I can’t just ask a lot of counter-questions.

#### Theme V: breaking the mutual silence

I just thought it was interesting how many [survey respondents] wanted information versus how many had received it.

According to HCPs, one important reason why conversations about sexual health do not occur in healthcare is that patients expect HCPs to initiate conversations if it is relevant to the patients’ health, and HCPs expect patients to raise the issue if they experience problems or have questions. Seeing the results from the survey, especially the difference between how many patients answered that they wanted or needed information and counselling and how many had received it caused several participants to react with shame and self-blame. But they also expressed that the results were expected and that they wanted it to be different.

It is clear that the patients want us to address it [sexual health] and I will try to get better at actually doing it.

Participants also stated that they try to address the subject with patients but that they probably need to be more direct in their approach.

It might require us to actually raise the question, because I think most of us, I sort of ask, is there something that you’ve been thinking about, something that you’d like to ask more about or is there something other than that which we have already addressed [that you want to talk about], and then they actually have the opportunity [to ask about sexual health] but they don’t. I think we need to take more of the initiative.

Breaking the silence required the normalisation of the subject and HCPs had to signal to patient that it was possible and encouraged to talk about it. Addressing the subject once wasn’t thought to be enough to break the silence. Asking questions several times and at different stages in rehabilitation was thought to increase the possibility for patients to express their need and wants.

It’s good that the more places the patient visits we [the HCP] address the same thing [the importance of sexual health], that we emphasise time and time again so that they understand that this is important and that we want to help with this.

Professionality was said to be important in making the subject talkable. Professionality in this context was described as making sexual health available, accessible by listening and taking it seriously, whereas shame and shyness were said to be unprofessional. Throughout the discussions on silence the relationship between HCP and patient was portrayed as being at the same time the barrier and the opportunity for breaking the mutual silence.

Well, there isn’t a concrete wall between me and my patient, it’s actually some other kind of wall […] this thing between me and my patient, something creates a barrier, and I know who it is. [it depends on me]

## Discussion

The results of this study highlight the impact of cardiac and respiratory conditions on patients’ sexual health and overall well-being, with nearly half indicating that sexual health has a large impact on their well-being. These findings align with existing literature, which emphasizes that chronic diseases affect sexual health, and that sexual health is important for overall well-being [8, 9]. Importantly, the study underscores a significant gap in sexual health counselling, with only 7% of patients reporting that they received information from healthcare professionals (HCPs), despite 84% expressing a desire for such information. This discrepancy points to a systemic challenge in healthcare communication and delivery.

The results also showed that HCPs’ experiences of providing sexual health information and counselling are shaped by individual factors such as assumptions about patients’ age, gender, and cultural background, as well as by organizational prerequisites like time constraints and lack of guidelines. Societal norms and varying levels of knowledge further influence whether and how these conversations occur, often resulting in sexual health being addressed inconsistently and primarily with male patients. Breaking the mutual silence and fostering professionalism, respect, and open communication are seen as essential steps to better support patients’ sexual health needs. This raises important questions about HCPs’ perceived ability and confidence in providing sexual health education. The tendency to focus on male patients, often through the lens of erectile dysfunction, may reflect a narrower understanding of sexual health that excludes the needs of women. This pattern suggests that HCPs may feel more equipped to address issues that are medically defined and treatable, while lacking the training or confidence to engage in broader, more nuanced conversations about sexuality, particularly with female patients.

A strength of this study is its focus on community-dwelling patients, which offers a valuable perspective on sexual health needs beyond traditional clinical environments. While most existing research has concentrated on hospital or outpatient settings, the sexual health concerns of individuals living in the community remain underexplored. This study highlights that such concerns are equally relevant in community contexts, where patients may encounter unique barriers to accessing information and counselling.

Understanding the differences between clinical and community care is essential to assess whether findings from one setting can be generalized to the other. For instance, primary healthcare plays a central role in the diagnosis and management of heart failure: half of all patients are diagnosed in primary care, and one-third are managed exclusively within it. These patients tend to be older, more often female, and more frequently affected by hypertension and pulmonary diseases, while less commonly presenting with ischaemic heart disease compared to patients within hospital settings [22, 23]. Community-dwelling patients may also have less frequent contact with healthcare professionals, rely more heavily on primary care or digital tools, and face a higher threshold for discussing sexual health. Recognizing these contextual differences is crucial for tailoring interventions and support mechanisms that are responsive to the specific needs of patients across diverse care settings.

Our results showed that men more frequently reported their disease affected their sexual health compared to women. Other studies suggest that this difference could be explained by various factors, including differences in disease perception, communication with HCPs, or societal norms regarding sexual health [24, 25]. This gendered pattern of communication ties directly to the experiences of the HCPs, which highlights the mutual silence between patients and HCPs. It suggests that HCPs may need to place greater emphasis on initiating sexual health discussions with women, who are often overlooked despite having significant concerns. Addressing this imbalance requires not only awareness but also targeted education and support for HCPs to feel confident in engaging with all patients, regardless of gender.

In our study, the low percentage of patients (7%) who received information on sexual health from HCPs is concerning, given the high demand for such information (84%). More than half of the patients expressed a desire to receive information on sexual health at various stages of their healthcare journey, and the preferred sources of information were predominantly HCPs.

Breaking the mutual silence between patients and HCPs was seen in this study as essential for effective sexual health communication. Patients often expect HCPs to initiate these conversations, while HCPs expect patients to bring up any issues [14]. The study’s findings indicate that HCPs need to be more proactive and direct in addressing sexual health, normalizing the subject through repeated discussions at various stages of care. The preferred settings for receiving sexual health information were both hospitals and primary care facilities, highlighting the importance of integrating sexual health discussions into routine medical consultations. This also reinforces the need for healthcare systems to facilitate these discussions in accessible and familiar environments, including community care settings [26, 27]. Younger patients (under 65 years) in this study showed a higher inclination towards receiving information and counselling on sexual health from HCPs and through mobile applications compared to older patients. Information and Communication Technology (ICT) appear to be underutilized in interventions aimed at improving sexual health among patients with chronic diseases. Most existing ICT focus on sexual dysfunction [28, 29] and not on sexual health as a whole. Future development of technology aiming to increase sexual health should be designed with input from both patients and HCPs to ensure usability and relevance. For HCPs, such tools could serve as conversation starters or educational aids during consultations, helping to normalize sexual health discussions and reduce discomfort. HCPs must be supported not only with knowledge but also with tools and time to apply it. This requires a shift in organizational culture and priorities.

The results also showed that HCPs felt their ability to provide sexual health counselling depended on the interplay between their own and patients’ identities and social positions, factors such as gender, age, and cultural background. Discomfort, lack of confidence, and patient factors such as cultural norms and values can be barriers to discussing sexual health [27, 30]. Self-reflection, routines, and openness could overcome these barriers [31]. Organizational factors such as time constraints, lack of support from management, absence of routines and guidelines, and insufficient resources can hinder the provision of sexual health information. HCPs may struggle to find adequate time during consultations to address sexual health comprehensively, and lack of support from management can hinder efforts to prioritize sexual health discussions [30]. Knowledge and experience in sexual health are important for HCPs to meet patients’ needs. Patients and HCPs emphasized the importance of creating opportunities for patients to discuss sexual health by asking direct questions and by fostering an open and respectful atmosphere. However, some HCPs questioned whether they possess the necessary knowledge and skills to provide optimal information and counselling about sexual health and there might be a need for standardized sexual education for HCPs [32]. A lack of adequate training regarding the connection between sexuality, sexual health and somatic health during undergraduate studies may contribute to insufficient competence in this area. This, in turn, can hinder HCPs ability to identify and understand patients’ needs, and may pose a barrier to ensuring patients’ rights to equitable and person-centred care. At the same time it should be recognized that opening the conversation to discuss sexual questions does not need expert knowledge, and the question that patients have are often of a very general nature and can be addressed by most HCPs [6, 7].

There are some methodological considerations in the study. First, the recruitment of patients through patient organizations, clinical outpatient departments, hospitals, and social media may introduce selection bias. Patients who are more engaged with patient organizations or active on social media might have different characteristics compared to the general population of patients with cardiac and respiratory diseases. Secondly, the HCPs were recruited from a single region in Sweden, which may limit the transferability of the findings to other regions or countries with different healthcare systems and cultural contexts.

## Conclusion

This study showed a significant gap between the sexual health needs of patients with cardiac and/or respiratory diseases and the information provided by HCPs. Most patients expressed a strong desire for more support and counselling on sexual health. HCPs identified several barriers to addressing these needs, including personal biases, organizational constraints, and a lack of knowledge. To bridge this gap, it is important for HCPs to adopt a proactive approach, ensuring respectful and clear communication to effectively address sexual health with their patients. To enable HCP to include sexual health in care, there is a need for organisational support such as guidelines, routines, and further training. This could not only improve patient well-being but also enhance the overall quality of care provided.

## Supplementary Information

Below is the link to the electronic supplementary material.

Supplementary Material 1

## Data Availability

Due to the nature of the sensitive personal data and study materials, data cannot be made freely available. However, by contacting the corresponding author, procedures for sharing data, analytic methods, and study materials for reproducing the results or replicating the procedure can be arranged following Swedish legislation.

## Abbreviations

HCPs: Healthcare Professionals
IQR: InterQuartile Range
CVD: Cardiovascular Disease
