# Supplementary material for: Information and counselling on sexual health in cardiac and respiratory disease: description of community dwelling patients’ needs and reflections of health care professionals
Source: Int J Equity Health. 2025 Nov 17;24:317. doi: 10.1186/s12939-025-02682-1 (PMC12621365; doi:10.1186/s12939-025-02682-1)
Supplement: Supplementary file 1 — Supplementary Material 1 [file 12939_2025_2682_MOESM1_ESM.docx]

**Supplementary material 1. Survey used to assess sexual health**

Advice and information about sexual health for patients with cardiovascular disease

Sexual health is important, regardless of health status.

The following questions are about sexual health and cardiovascular and lung disease. The aim of this survey is to investigate access to information and support regarding sexual health for patients with cardiovascular disease. There are many connections between cardiovascular diseases and sexual health, which is why we are interested in your experiences and needs regarding information and counseling.

This survey has been designed by the Division of Nursing Sciences and Reproductive Health, at the University of Linköping together with the Knowledge Centre for Sexual Health in Region Västra Götaland (Kunskapscentrum för sexuell hälsa).

Your responses are anonymous, and the results will be presented at a group level so that no individual can be identified. The survey takes about 10 minutes to complete. Choose the response options that best apply to you for each question. Please note that for some questions, you can provide more than one answer. We need your responses by March 31, 2024.

**Conditions, diseases, and treatments - What applies to you?**

(Please note that more than one answer is possible)

- Heart attack
- High blood pressure
- Angina
- Heart failure
- Blood clot (Thrombosis)
- TIA
- Stroke
- Aortic aneurysm
- Atrial fibrillation
- Valve disease
- Arrhythmia
- Congenital heart disease
- Pacemaker
- Defibrillator
- Cardiac surgery
- Lung disease such as asthma or COPD
- Sudden cardiac arrest

**Sexual health**

Sexual health encompasses aspects such as sexuality, body and function, health status, pleasure, and satisfaction. Good sexual health can be achieved whether you engage in sexual activities with yourself or with others, and even if you choose not to have sex at all and are content with that. To take care of your sexual health, access to information, healthcare, and counseling is necessary.

**Do you feel that your sexual health affects your well-being?**

- Not at all
- To some extent
- To a large extent
- To a very large extent

**Do you feel that your sexual health has been affected by your diagnosis, illness or treatment?**

- Not at all
- To some extent
- To a large extent
- To a large extent
- My sexual health has been affected in a positive way

**Experiences of receiving information and counselling on sexual health from the healthcare system.**

**Have you received information or counseling from a healthcare professional about how your sexual health may be affected by your illness, diagnosis, or treatment?**

- Yes
- No

**I have received information or counseling on:**

(Multiple answers are possible)

- Erectile dysfunction
- Pain during sex
- Anxiety or fear about sexual activity
- Side effects from drugs
- Side effects and complications of procedures
- When you can have sex, with yourself or with others
- Lust, desire and relationships

**When did you receive information or counseling about sexual health?**

(Multiple answers are possible)

- In connection with diagnosis
- In connection with drug prescription
- Before surgery
- During follow-up after surgery
- During follow-up of prescription of medication
- At an annual check-up

**Who provided you with information or counseling about sexual health?**

(Multiple answers are possible)

- Social worker/Counselor/Psychologist
- Nurse
- Doctor
- Physiotherapist
- Occupational therapist
- Assistant nurse
- Dietician
- Chat or phone call with 1177
- Don’t know/can’t remember

**Where did you get information or counseling about sexual health?**

(Multiple answers are possible)

- Primary care center (Vårdcentral)
- Rehabilitation centre
- Hospital or clinic
- Online

**Did you wish or need to receive information or counseling about sexual health?**

- Yes
- No
- Don’t know

**Have you received information or counseling about how your sexual health may be affected by your illness/diagnosis/treatment from someone outside of healthcare?**

- Yes
- No

**Who gave you information or advice?**

(Multiple answers are possible)

- Patient organisation, e.g., Hjärt-Lung foundation or Heart and Lung Patient organisation
- From the national care platform 1177
- From other source via the internet
- From an NGO promoting sexual and reproductive health and rights, e.g., RFSU or RFSL

**What information and advice about sexual health would you have wanted or needed?**(Multiple answers are possible)

- Erectile dysfunction
- Pain during sex
- Anxiety or fear about sexual activity
- Impact on relationships, lust and desires
- Side effects of drugs
- Side effects and complications of procedures
- When it is safe to have sex, with yourself or with others
- Sexual practices that involve high levels of stress or strain

**Have you been able to ask questions to healthcare professionals about sexual health?**

- Yes
- No
- Don’t know

**When do you think it would be best to receive information, counseling and advice?**
(Multiple answers are possible)

- In connection with diagnosis
- In connection with drug prescription
- Before surgery
- During follow-up after surgery
- During follow-up of prescription of drugs
- At an annual check-up

**Who do you think should provide the information and be able to answer questions about sexual health?**
(Multiple answers are possible)

- Social worker/Counselor/Psychologist
- Nurse
- Doctor
- Physiotherapist
- Occupational therapist
- Assistant nurse
- Dietician
- Chat or phone call with 1177
- Don’t know

**Where do you think it should be possible to receive information and ask questions about sexual health?**

(Multiple answers are possible)

- Primary care center (Vårdcentral)
- Rehabilitation center
- Hospital or clinic

**How do you want to receive information about sexual health?**

(Multiple answers are possible)

- Conversations with healthcare professionals
- Discussion groups
- Brochure
- On the national care platform 1177
- On patient organisations website
- Information film
- App
- Opportunity to receive information and counseling from various sources at various times, e.g., at diagnosis, drug prescription and before surgery.

**How do you think partners should receive information and advice about sexual health in relation to cardiovascular disease?**

(Multiple answers are possible)

- Not at all
- Verbally from healthcare professionals without me being present
- In conversation with healthcare professionals together with me
- Written information
- Via discussion groups

**For those providing information, counseling or advice about sexual health, to patients with cardiovascular disease, what is important to consider?**

**Questions about you**

(Note that all your answers are anonymous and that the result will be presented at a group level so that no individual can be identified)

**How old are you? _______ years old**

**What is your gender?**

- Woman
- Man
- Nonbinary

**Are you transgender or do you have trans experiences?**

- Yes
- No

**Additional comments, suggestions and feedback**

**Please share any other comments, suggestions and feedback:**
